# Predicting skilled delivery service use in Ethiopia: dual application of logistic regression and machine learning algorithms

**DOI:** 10.1186/s12911-019-0942-5

**Published:** 2019-11-05

**Authors:** Brook Tesfaye, Suleman Atique, Tariq Azim, Mihiretu M. Kebede

**Affiliations:** 1 World Health Organization, Kenya Country Representative Office, United Nations Office in Nairobi (UNON), Gigiri Complex, Block “U”, Nairobi, Kenya; 2 Department of Health Informatics, University of Ha’il, College of Public Health and Health Informatics, Hail, Kingdom of Saudi Arabia; 3 Faculty of Allied Health Sciences, Institute of Public Health, University of Lahore, Lahore, Pakistan; 4 John Snow Incorporated (JSI) Research and Training Institute, Arlington, VA USA; 5 Leibniz Institute for Prevention Research and Epidemiology –BIPS, Achterstraße, 30 Bremen, Germany; 6 University of Bremen, Health Sciences, Bremen, Germany; 7 Department of Health Informatics, University of Gondar, College of Medicine and Health Sciences, Institute of Public Health, Gondar, Ethiopia

**Keywords:** Skilled delivery, Machine learning

## Abstract

**Background:**

Skilled assistance during childbirth is essential to reduce maternal deaths. However, in Ethiopia, which is among the six countries contributing to more than half of the global maternal deaths, the coverage of births attended by skilled health personnel remains very low. The aim of this study was to identify determinants and develop a predictive model for skilled delivery service use in Ethiopia by applying logistic regression and machine-learning techniques.

**Methods:**

Data from the 2016 Ethiopian Demographic and Health Survey (EDHS) was used for this study. Statistical Package for Social Sciences (SPSS) and Waikato Environment for Knowledge Analysis (WEKA) tools were used for logistic regression and model building respectively. Classification algorithms namely J48, Naïve Bayes, Support Vector Machine (SVM), and Artificial Neural Network (ANN) were used for model development. The validation of the predictive models was assessed using accuracy, sensitivity, specificity, and area under Receiver Operating Characteristics (ROC) curve.

**Results:**

Only 27.7% women received skilled delivery assistance in Ethiopia. First antenatal care (ANC) [AOR = 1.83, 95% CI (1.24–2.69)], birth order [AOR = 0.22, 95% CI (0.11–0.46)], television ownership [AOR = 6.83, 95% CI (2.52–18.52)], contraceptive use [AOR = 1.92, 95% CI (1.26–2.97)], cost needed for healthcare [AOR = 2.17, 95% CI (1.47–3.21)], age at first birth [AOR = 1.96, 95% CI (1.31–2.94)], and age at first sex [AOR = 2.72, 95% CI (1.55–4.76)] were determinants for utilizing skilled delivery services during the childbirth. Predictive models were developed and the J48 model had superior predictive accuracy (98%), sensitivity (96%), specificity (99%) and, the area under ROC (98%).

**Conclusions:**

First ANC and contraceptive uses were among the determinants of utilization of skilled delivery services. A predictive model was developed to forecast the likelihood of a pregnant woman seeking skilled delivery assistance; therefore, the predictive model can help to decide targeted interventions for a pregnant woman to ensure skilled assistance at childbirth. The model developed through the J48 algorithm has better predictive accuracy. Web-based application can be build based on results of this study.

## Background

Reducing the global Maternal Mortality Ratio (MMR) to less than 70 deaths per 100,000 births is one of the targets of the Sustainable Development Goals (SDGs). In addition, a secondary objective of this goal is to have no country with a MMR more than twice the global average [1]. However, maternal mortality persists to be high; with about 830 women dying due to complications during and following pregnancy and childbirth each day. In 2015, an estimated 303,000 women died due to maternal causes, with 99% of these deaths, most of which could have been averted, occurring in developing countries [2, 3]. Major complications that account for nearly 75% of all maternal deaths are obstructed labor, ruptured uterus, severe pre-eclampsia/eclampsia, malaria and, complications from abortion [4–7].

Skilled care before, during, and after childbirth can save the lives of both women and newborns [8, 9]. The highest number of maternal deaths occurs due to complications on the first 24 h after childbirth, indicating the importance of quality care during early postnatal period [10–16]. Most of these complications, and thus significant number of maternal mortalities and morbidities, could have been prevented with the presence of a skilled birth attendant at the time of childbirth [12, 14, 17].

More than half of the global maternal deaths occurred in high-burden countries, Ethiopia being one of them with an estimated MMR of 412 per 100,000 live births [3, 18–20]. Home delivery, without the presence of a skilled birth attendant (defined as a midwife, trained nurse, doctor, or a health extension worker/community health worker) is unacceptably high in Ethiopia [20, 21] . Skilled delivery is one of the most monitored indicators of the Millennium Development Goals (MDGs). Skilled assistance before, during, and after childbirth could avert up to three-quarter or more of maternal deaths [22, 23]. However, coverage of skilled delivery remained low, although rapid expansion of health facilities and trained human resources are being improved. In Ethiopia, the proportion of births attended by Skilled Birth Attendance (SBA) in 2000, 2005, 2011 and 2016 was about 6.0, 5.7, 10.0, and 27.7% respectively [20]. Various determinants that influence skilled delivery service use or delivery at home have been well studied [24]. Knowledge from these studies can be applied to develop models that can help to predict the likelihood of a woman using skilled delivery services, and thereby, can help to target specific interventions for that woman to ensure that she is provided skilled services during childbirth.

Machine-learning is a novel method of analyzing big databases, exploring meaningful information and developing models for prediction, clustering and associations [25–28]. Machine-learning algorithms are methodologies used for big data analytics. These algorithms make data mining capable of mining information that commonly used statistical methods (logistic regression) fail to present. While statistical methods only quantify data, data mining develops models to identify hidden patterns and relationships in the data, usually from large volumes of database. Machine-learning is a widely used method in computing which is becoming popular in medicine and public health as well [29]. Large number of studies have identified predictors of skilled delivery service use. However, they fail to present information that is unknown in advance. Applying data mining techniques is different from commonly used statistical methods (for example logistic regression) as it extracts valuable information on the absence of any clear hypothesis [30]. Furthermore, large volumes of quality data, such as DHS, are being collected and recorded in the health sector. There is a growing need of applying better analytical techniques on those voluminous data to extract evidences that improve decisions in the health sector. In this study, we aimed to assess determinants of skilled delivery use by applying a reliable predictive model using machine-learning methods.

## Methods

### Study setting, design and period

The study was conducted in Ethiopia from May to December 2018. A secondary data analysis was applied using data from the recent Ethiopian Demographic and Health Survey (EDHS) of 2016.

### Study population

Women in the reproductive age group (aged 15 to 49 years) who gave at least one live birth in the last five years preceding the survey were included in this study.

### Study design and sampling techniques

For EDHS 2016, 18,008 households were sampled and the survey was implemented in 17,067 households. From these households 16,583 eligible women were interviewed with response rate of 94.2%. Of these women, 11,023 women belong to the age group 15–49 years, and had given at least one birth in the last five years preceding the survey.

### Data collection instruments and procedures

Data on the utilization of skilled delivery services and associated characteristics was collected from the study population administering a Demographic and Health Surveys (DHS) standardized women’s questionnaire [31]. However, if the woman had more than one child in the last five years preceding the survey, information on the use of skilled assistance during delivery was collected for the last birth [31–33].

### Data analysis and presentations

Data cleaning was performed to prepare the dataset for analysis based on objectives of the study.

Study variables were re-coded to meet the desired classification. To overcome unbalanced distribution of samples [34] and ensure representativeness of survey results at national level [35], sampling weights were used during analysis. Twenty-seven records (0.24%) with missing values were excluded from the analysis to improve the quality of predictive models being developed. Statistical Package for Social Sciences (SPSS) for Windows version 23.0 software was used for the analysis. Descriptive statistics was carried out to describe women’s characteristics. Bivariate analysis was conducted to investigate association of dependent and independent variables. All variables that show a statistically significant association (*p* < 0.05) on bivariate analysis were entered into multivariable logistic regression analysis to control the effect of confounding factors. To estimate the effect of the independent variables on the dependent variable, Odds Ratio (OR) with its 95% Confidence Interval (CI) were computed.

### Predictive modeling

Predictive model development was based on variables that were found significant in the multivariable logistic regression analysis. Data about the study population based on those variables was extracted. Waikato Environment for Knowledge Analysis (WEKA) for Windows version 3.8.1 software was utilized to conduct the predictive modelling.

Classification method of machine learning was performed by organizing the data in given classes [36]. This classification method, known as supervised classification, uses given class labels to order the objects in the data collection where all objects are already associated with known class labels. The classification algorithm learns from the training dataset and builds a model, which is used to classify new objects, known as test dataset [37]. Synthetic Minority Oversampling Technique (SMOTE) was applied to balance the dataset and minimize sampling errors [36]. Information-gain attribute selection method in WEKA [36] was applied to rank attributes having strong association with skilled delivery. This helped to classify the database and developing models with highest accuracy possible. As a result, seven attributes identified from the multivariable logistic regression analysis (Table 2) were considered for developing predictive model.

Estimating performance of classifiers, a stratified ten-fold cross-validation approach, was used. Empirical studies exhibited that the ten-fold cross-validation seem to be an optimal number of folds (that optimizes the time it takes to complete the test while minimizing the bias and variance associated with the validation process) [38, 39]. In the ten-fold cross-validation, the entire dataset was divided into ten mutually exclusive folds with approximately the same class distribution as the original dataset. Each fold was used once to test the performance of the classifier that is generated from the combined data of the remaining nine folds, leading to ten independent performance estimates.

The dataset was randomly categorized into two groups, training and test groups. The training group comprised of 9920 cases (90% of the whole dataset). The prediction model was developed relying on the training group. The remaining 1103 cases (10% of the whole dataset) were allocated as the test group for model evaluation [40]. Model training was conducted frequently. Since a large sample was undertaken, 90/10% split rule was applied for model development and testing [41].

In this study, four classification algorithms namely Naïve Bayes, J48, Artificial Neural Network (ANN), and Support Vector Machine (SVM) classification algorithms in WEKA were employed. Accordingly, in line with maternal health domain expert’s recommendation, the study developed four sub models namely sub model I, II, III and IV. The proposed models predicted whether pregnant women would utilize skilled delivery services or not. All sub models were developed using all input variables and derivation dataset. The performance of learning algorithms was compared based on the conventional predictive accuracy measures i.e. accuracy, area under the Receiver Operating Characteristic (ROC) curve, sensitivity and specificity [42]. The prediction performance of each class was measured separately in addition to the overall accuracy of all classes. In the contingency table, the rows represent the actual class, and the columns represent the predicted class. Each number in the contingency table is the number of records in the databases corresponding to the predicted class and the actual class [42]. It is essential to construct an interpretation of developed models [25]. Thus, interesting rules were extracted from outperforming predictive model and demonstrated using IF-THEN statements that were displayed in a decision tree.

## Results

### Socio-economic and demographic characteristics of the study population

Eleven thousand twenty-three women aged 15–49 who gave a live birth in the last five years preceding the survey were included in the study. The mean age of the mothers was 29.23 (SD + 6.54), nearly 55.0% of whom were below the age of 29. As indicated in Table 1, the majority of the respondents were rural (89.0%) residents. More than 82% of the mothers in the overall sample had never attended a formal education. The mean family size of the study population was 6.06 (SD + 2.19) persons.

**Table 1:** Socio-economic and demographic characteristics of the study population in Ethiopia in 2016 (*n*=11,023)

Characteristics	Skilled assistance during delivery		Total number of births (n)
	Yes (%)	No (%)	
Region			
Tigray	59.2	40.8	716
Afar	16.5	83.5	115
Amhara	27.8	72.2	2072
Oromia	19.6	80.4	4850
Somali	20.1	79.9	508
Benishangul	28.7	71.3	122
SNNPR	28.6	71.4	2296
Gambela	48.1	51.9	27
Harari	50.0	50.0	26
Addis Adaba	96.7	3.3	244
Dire Dawa	57.4	42.6	47
Place of residence			
Urban	80.1	19.9	1215
Rural	21.2	78.8	9807
Family size			
Five or less	35.7	64.3	4847
Six and above	21.4	78.6	6176
Age of mother's in years			
15 - 19	38.9	61.1	378
20 - 34	29.1	70.9	7910
35 - 49	22.0	78.0	2735
Birth order			
1	37.0	63.0	4078
2 - 4	22.4	77.6	6878
5+	4.5	95.5	67
Current marital status			
Never Married	63.2	36.8	57
Married/Living together	27.3	72.7	10462
Separated/Widowed/Divorced	32.8	67.2	504
Maternal education			
No Formal Education	17.2	82.8	7284
Primary	38.6	61.4	2951
Secondary and above	83.5	16.5	788
Wealth status			
Poor	15.8	84.2	5156
Medium	24.3	75.7	2280
Rich	47.0	53.0	3587
Occupation of mother's			
Not Working	25.2	74.8	6127
Working	30.8	69.2	4896
Women’s decision making on their own healthcare			
Independent	35.1	64.9	1362
Dependent	26.7	73.3	9661
First antenatal care (n=7590)			
Yes	88.79	11.21	2524
No	49.21	50.79	5066

Forty six percent (46.8%) of the respondents were in the poor wealth quintile (Table 1). Nearly two-third, 68.8%(7590), of the respondents who had a live birth in the five years preceding the survey received first antenatal care.

### Determinants of skilled delivery

In EDHS 2016, of the total 15,683 women aged 15–49 were included in the survey, 11,023 (70.2%) of them gave at least one live birth within five years preceding the survey. Among them, only 3053 (27.7%) proportion of the births received skilled assistance during delivery.

Table 2 presents the results of the analysis of determinants of skilled delivery. After adjusting for confounders, higher birth order, television ownership, cost needed for healthcare, contraceptive use, age at first sex, and age at first birth, first antenatal care were significantly associated with skilled delivery service use.

**Table 2:** Bivariate and multivariate logistic regression for the association between independent variables and skilled delivery service utilization in Ethiopia in 2016 (*n*=11,023)

Characteristics	Skilled assistance during delivery		Crudes OR (95% CI)	Adjusted OR (95% CI)	*p* - value
	Yes	No			
Birth order					
1	1026 (49.8%)	1033 (50.2%)	1	1	
2 - 3	1042 (31.0%)	2317 (69.0%)	0.45 (0.40 - 0.51)**	0.66 (0.38 - 1.14)*	0.042
4 - 5	498 (19.1%)	2108 (80.9%)	0.24 (0.21 - 0.27)**	0.22 (0.11 - 0.46)**	0.000
6+	487 (16.2%)	2514 (83.8%)	0.19 (0.17 - 0.22)**	0.39 (0.17 - 0.91)*	0.030
Television ownership					
Yes	809 (85.1%)	142 (14.9%)	19.92 (16.56 - 23.94)**	6.83 (2.52 - 18.52)**	0.000
No	2244 (22.3%)	7829 (77.7%)	1	1	
Cost needed for healthcare					
Not a big problem	1579 (37.9%)	2583 (62.1%)	2.24 (2.05 - 2.43)**	2.17 (1.47 - 3.21)**	0.000
Big problem	1473 (21.5%)	5387 (78.5%)	1	1	
Modern family planning					
User	1440 (41.8%)	2009 (58.2%)	2.06 (1.76 - 2.42)**	1.92 (1.26 - 2.97)**	0.003
Non user	1613 (21.2%)	5981 (78.8%)	1	1	
Age at first sex					
19 or below	2317 (25.2%)	6890 (74.8%)	2.02 (1.82 - 2.25)**	2.72 (1.55 - 4.76)**	0.000
20+	735 (40.5%)	1080 (59.5%)	1	1	
Age at first birth					
19 or below	1521 (23.9%)	4843 (76.1%)	1	1	
20+	1414 (35.6%)	2558 (64.4%)	1.76 (1.61 - 1.92)**	1.96 (1.31 - 2.94)**	0.001
Received first antenatal care					
Yes	2241 (47.3%)	2493 (52.7%)	8.17 (7.13 - 9.35)**	1.83 (1.24 - 2.69)**	0.002
No	283 (9.9%)	2573 (90.1%)	1	1	

*Statistically significant at *p*-value < 0.05

**Statistically significant at *p*-value < 0.01

Television ownership [AOR = 6.83, 95% CI (2.52–18.52)] had the strongest associations with skilled delivery service use. The probability of skilled delivery service use was reduced with increasing birth order. The likelihood of skilled delivery service utilization decreased by approximately 78% in the last-born in the household compared to the first-ordered child [AOR = 0.22, 95% CI (0.11–0.46)] in the household.

Age at first sex [AOR = 2.72, 95% CI (1.55–4.76)] was positively associated with skilled delivery service utilization, as women who had their first sex at the age of nineteen or below had almost a threefold increase in the probability of skilled delivery service utilization.

The likelihood of skilled delivery service utilization had a positive correlation with the age at first birth and women who had received first antenatal care. Women age at first birth of twenty and above had almost a twofold increase [AOR = 1.96, 95% CI (1.31–2.94)] in the probability of skilled delivery service use compared to their counter parts.

Women who had received first ANC had almost a two-fold increase in the odds of skilled delivery service use compared to those women who never received first antenatal care [AOR = 1.83, 95% CI (1.24–2.69)].

Women who can afford healthcare had a two-fold increase in the probability of skilled delivery service use than those who could not afford [AOR = 2.17, 95% CI (1.47–3.21)]. The probability of skilled delivery service use increased with women who used contraceptives [AOR = 1.92, 95% CI (1.26–2.97)] as compared to those who were non-users.

Those women who live in a television-equipped household were 6.83 times [AOR = 6.83, 95% CI (2.52–18.52)] more likely to receive skilled assistance during delivery when compared to their counterparts.

### Predictive modeling

In the information gain attribute selection analysis (Table 3), television ownership, first antenatal care visit during pregnancy, and birth order were found to be the first three most important attributes in predicting skilled delivery service utilization. Thus, experiments were undertaken in order to develop a predictive model.

**Table 3:** List of attributes ranked according to information gain attribute selection technique

Variable name	Type	Information gain score	Rank
Television ownership	Nominal	0.15	1
First antenatal care	Nominal	0.15	2
Birth order	Nominal	0.06	3
Contraceptive use	Nominal	0.05	4
Cost needed for healthcare	Nominal	0.04	5
Age at first birth	Nominal	0.02	6
Age at first sex	Nominal	0.01	7

Among all sub-models, the model developed with J48 had better performance measures, accuracy, sensitivity, AUC and Positive Predictive Value (PPV) (Table 5). The model performed at an accuracy of 98%. This implies that among all 14,188 instances (after SMOTE), 13,904 (98%) were correctly classified whereas the remaining 284 (2%), were incorrectly classified (Table 4).

**Table 4:** Contingency table for classification of skilled delivery service utilization based on sub model II

		Predicted class	
		No skilled delivery	Yes skilled delivery
Actual class	No-skilled -delivery	701	17
	Yes skilled delivery	36	675

Of the total 14,188 instances, the model had scored Positive Predictive Value (PPV) of 99.9% and Negative Predictive Value (NPV) of 96.1%. The sensitivity and specificity of the model were 96.2 and 99.9% respectively. The areas under ROC (AUC) and F-measure were both 98.0% (Table 5).

**Table 5:** Accuracy analysis of skilled delivery prediction sub-models in Ethiopia in 2016

Sub model	Algorithm	Accuracy	Sensitivity	Specificity	AUC	PPV	NPV	Time (in Seconds)
I	J48	98.0%	96.2%	99.9%	98.0%	99.9%	96.1%	0.09
II	ANN	97.8%	98.4%	99.8%	98.6%	99.8%	95.4%	8.91
III	SVM	97.8%	94.8%	99.9%	97.8%	99.9%	95.5%	15.94
IV	Naïve Bayes	96.9%	95.1%	97.5%	98.8%	97.6%	94.9%	0.02

### Some rules extracted from the decision tree

Architecture of outputs from the decision tree was developed based on the outperforming sub-model I and was large and neatly visible. However, some rules extracted from the decision tree were:

1. IF a woman gives birth at a health facility and does not received first antenatal care THEN she will not receive skilled assistance during delivery.
2. IF a woman gives birth out of a health facility, receives first antenatal care, child is second or third-born, and does not have a television THEN she will not receive skilled assistance during delivery.
3. IF a woman gives birth out of a health facility, receives first antenatal care, child is second or third-born, owns a television, and had her first sex at age of 20 or above THEN she will receive skilled assistance during delivery.
4. IF a woman gives birth at a health facility, receives first antenatal care, child is second or third-born, had cost problem for healthcare, and had her first sex at age of 20 or above THEN she will not receive skilled assistance during delivery.

## Discussion

The present study demonstrated determinants of skilled delivery in Ethiopia using a commonly used statistical method (logistic regression) and developed a predictive model to forecast skilled delivery service use by applying machine-learning algorithms using data from a nationally representative survey in Ethiopia.

Prediction machine-learning methods are becoming popular and increasingly important in healthcare research due to their ability to handle large databases that are becoming available. This study has demonstrated relevance of machine-learning algorithms to develop prediction models based on attributes found to be determinants in multivariate logistic regression analysis.

All classification algorithms deployed in the study were substantially accurate to predict skilled delivery service use and the results were more profound [25] compared to results from commonly used statistical methods. This is because data mining algorithms reveal undiscovered relationships and interactions between attributes in the DHS data that improve the prognosis of skilled delivery use. A model developed using J48 algorithm had the highest prediction accuracy. This result indicated that J48 algorithm is the most appropriate algorithm deployed in this study to classify records of the database and make the most precise prediction. In addition, results of the J48 algorithm have the advantage of being more easily interpretable, especially when compared with closed models, called black box models, such as ANN. This advantage makes it a commonly used algorithm by the medical community [42, 43].

This study further ranked the most important attributes for predicting skilled delivery service utilization in Ethiopia which are (ordered most to least important) television ownership, first antenatal care, birth order, modern family planning, cost needed for healthcare, age at first birth, and age at first sex. Eventually, the developed predictive models at a programmatic level can help to identify the most influential determinants to promote utilization of skilled delivery services by pregnant women and thereby support evidence-based decision making in maternal health intervention programs in Ethiopia. On the other hand, at a service provider level, such models can be incorporated as a routine health service delivery tool for pregnant women in order that, based on the attributes of the pregnant woman, the service provider can identify the likelihood of a women availing skilled services at the time of delivery. In that way, the service provider can provide personalized health education and motivate the pregnant woman and her family members and the “Health Development Army (HDA)” team leaders in the Ethiopian health system that ultimately improve the use of skilled delivery services.

The study revealed that the proportion of births attended by SBA in Ethiopia in 2016 was very low, as justified in previous studies [2, 36, 43], even compared to some low-income countries [44–46]; only 27.7% of the women received skilled assistance during delivery [20].

Birth order, first antenatal care, modern family planning, cost needed for healthcare, age at first birth, age at first sex, and television ownership were found to have significant association with skilled delivery.

Most studies in Ethiopia and other parts of the world have found a strong association between first antenatal care and skilled delivery service use [47–49]. The findings in this study corroborated that the likelihood of woman in Ethiopia receiving a skilled assistance during delivery is higher for women who have had received first antenatal care during pregnancy. This may be attributed to the fact that first antenatal care has the potential to better create awareness on the benefits of institutional delivery [50] and inform relevant knowledge about dangerous signs of pregnancy and safe delivery care [18, 24, 51–54]. On the contrary, a study conducted in Kenya reported that the use of institutional delivery services was very low even among antenatal care attendees [55].

The finding that skilled delivery service utilization is associated with deliveries conducted in health facilities is in line with the expectation and is reported by other studies [2, 24, 50]. Skilled assistance during institutional delivery is very common [24, 53], and studies have reported that of all skilled assistance deliveries, majority of them were conducted at health facilities [56].

These findings give credence to the differential in birth order in the likelihood of skilled delivery service utilization as well. The study results revealed that the likelihood of skilled delivery service utilization decreased with increasing birth order. This is in agreement with findings from previous studies in the same setting [56–58] and other parts of the world such as India [24, 59, 60]. The first birth is known to be more difficult [61] and a woman who had no experience of delivery are evidently more likely to use skilled birth attendance compared to any of the birth order categories [57] and in some settings the woman’s family helps her get the best care possible [48]. On the other hand, women who had safe maternity experiences might neglect the need to have skilled assistance [60] even though very high-order births are more risky [60–62].

The study results highlight women who owned television were more likely to utilize skilled assistance during delivery as compared to those who did not own. This may be because mass media tools like television have a potential to disseminate information that geared towards promoting health-related behaviors including skilled delivery service utilization [32, 63, 64].

Furthermore, the study found that the cost needed for healthcare was a major constraint in skilled delivery service utilization. Different studies in Ethiopia [45, 65] and across the globe have upheld similar findings. Even though, in Ethiopia, maternal health services are given free of charge by law, there is a policy practice gap in the implementation of the law [66] and mothers may not be well aware. In addition, there are hidden indirect costs such as transportation [24, 35], lodging expenses [61], and costs related to treatment at the facility [67–71].

### Limitations of the study

Our study utilized secondary data. Thus, authors were unable to conduct further analysis on key contexts such as maternal deaths. Authors are not privy to how low the response rate was, how cases were excluded due to poor data quality, and the how the recall bias affected the data, and thus the findings. Similar studies, following similar methodology, were scarce, as to the knowledge of the authors.

## Conclusions

Birth order, first antenatal care, modern family planning, cost needed for healthcare, age at first birth, age at first sex, and television ownership were found to be determinants of skilled delivery use in Ethiopia.

Among the prediction models developed using J48, ANN, SVM, and Naïve Bayes data mining algorithms, J48 algorithm had a relatively more accurate predictive performance. The results of this study can be useful for developing a web-based application. Experimentation with other classification algorithms for skilled delivery service use as well as other maternal health services will help to develop the most accurate models.

## Data Availability

The data used for this study is available on MEASURE DHS website (https://dhsprogram.com/data/) and can be accessed with permissible application.

## Abbreviations

ANN: Artificial Neural Network
DHS: Demographic and Health Survey
EDHS: Ethiopian Demographic and Health Survey
ROC: Receiver Operating Characteristics
SBA: Skilled Birth Attendance
SVM: Support Vector Machine
